# Extended multisystem manifestations of hereditary α-tryptasemia in an allergy center cohort

**DOI:** 10.1016/j.jacig.2026.100737

**Published:** 2026-05-20

**Authors:** Daniel Koch, Friederike Wortmann, Andreas Recke, Ellen Rose, Evelyn Gaffal, Jennifer Wigger, Nikolas von Bubnoff, Dagmar von Bubnoff

**Affiliations:** a Department of Dermatology, Allergology, and Venerology, University Hospital Schleswig-Holstein, Lübeck, Germany; b Department of Hematology and Oncology, University Hospital and University Cancer Center Schleswig-Holstein, Lübeck, Germany

**Keywords:** Hereditary α-tryptasemia, *KIT*^D816V^ mutation, tryptase, phenotype, symptom burden, multisystemic, allergy

## Abstract

**Background:**

Hereditary α-tryptasemia (HαT) is a common autosomal-dominant trait caused by additional copies of the gene *TPSAB1* encoding for α-tryptase. This inheritance is the most common etiology for elevated basal serum tryptase (BST), occurring almost exclusively at BST ≥ 8 μg/L. In systemic mastocytosis (SM) and nonclonal mast cell–mediated disorders, HαT is linked to a complex constellation of symptoms.

**Objective:**

We sought to delineate the spectrum and burden of extended manifestations observed in patients with and without HαT presenting to an allergy department.

**Methods:**

A total of 332 allergy patients with BST ≥ 8 μg/L were prospectively enrolled. Blood was tested for HαT and *KIT*^D816V^ mutation (to identify patients with SM) by digital droplet PCR. Symptom assessment was conducted with a HαT-customized questionnaire (n = 256).

**Results:**

HαT-positive patients reported a significantly higher symptom burden than HαT-negative patients (9.3 vs 6.5; *P* < .001), particularly those referred for urticaria/angioedema (8.9 vs 5.5; *P* = .023) and atopic diseases (8.3 vs 4.2; *P* = .016). In allergy patients with HαT, symptom spectrum was extended to fatigue (+26%; *P* < .001), sleep disturbances (+13%; *P* = .049), and neuropsychiatric symptoms such as concentration issues (+26%; *P* < .001), memory impairment (+16%; *P* = .020), sadness/lack of motivation (+22%; *P* = .004), headaches (+19%; *P* = .006), abdominal pain (+21%; *P* = .002), and cardiovascular symptoms such as dizziness (+14%; *P* = .052), palpitations (+15%; *P* = .039), and orthostatic dysregulation (+22%; *P* = .002).

**Conclusion:**

HαT may have a broader clinical impact beyond classic allergy–immunology connections and may be associated with extended clinical phenotypes. This suggests an integrative approach to medical practice in allergy patients.

## Graphical abstract

**Figure:**
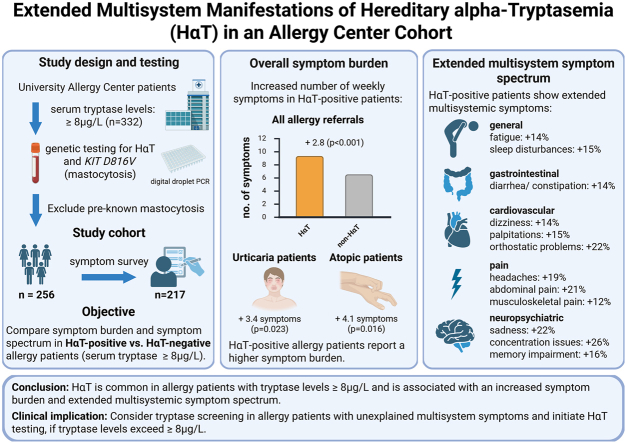

Baseline serum tryptase (BST) levels elevated beyond the reference value of BST < 11.5 μg/L are common and are estimated to occur in 6% to 7% of the general population.^1^ They indicate an increased risk of severe anaphylaxis to, for example, Hymenoptera venom, drugs, or food.^2^ Hereditary α-tryptasemia (HαT), with its prevalence of 5% to 7% in our Western society, has been shown to be the main cause of elevated BST in the general population.^3^, ^4^, ^5^, ^6^ HαT is almost exclusively found in individuals at BST ≥ 8 μg/L (at high-normal BST from 8 to 11.4 μg/L and elevated BST ≥ 11.5 μg/L).^3^^,^^4^ The tryptases released from mast cell granules determine the BST and consist of α-isoform tryptases of the gene locus *TPSAB1* and the β-isoform tryptases of the gene loci *TPSAB1* and *TPSB2*. *TPSB2* only encodes for β-tryptase, whereas *TPSAB1* either encodes for α- or β-tryptase. In an individual, the genotypes of tryptase are determined on the basis of whether *TPSAB1* encodes for α- or β-tryptase. This results in different genotypes of tryptase (Fig 1). HαT occurs only in individuals who carry an α-tryptase encoding gene, *TPSAB1,* and in whom this gene is duplicated or multiplied on the same allele.^3^^,^^7^ Fig 1 summarizes possible haplotypes and genotypes in a wild-type gene for *TPSAB1* and for HαT. A gain of α copies increases BST levels by approximately 9 μg/L per copy.^7^ Most individuals with HαT (>90%) have only one additional α-tryptase copy (eg, αα/β, β/β) with a median BST slightly above the reference value (13.6 μg/L; reference, <11.5 μg/L). Individuals with 2 additional α copies (eg, ααα/β, β/β) have a median BST of 22.5 μg/L.^7^

**Fig 1:**
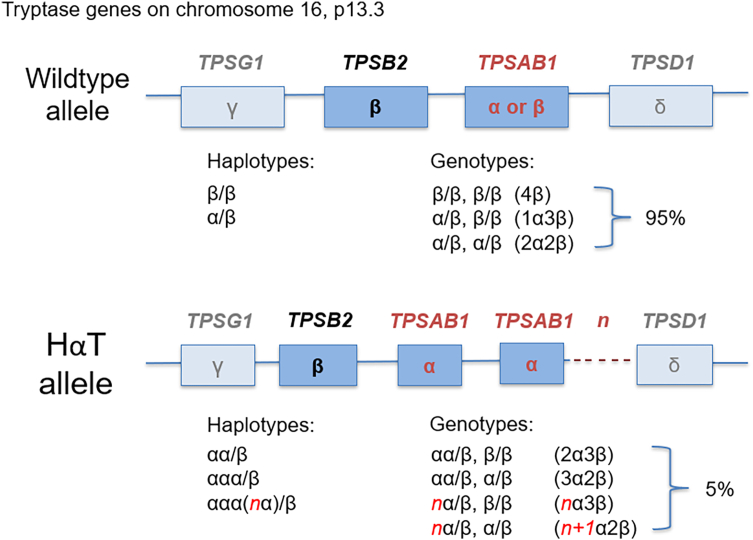
Wild-type and HαT alleles on chromosome 16. Possible haplotypes and genotypes with prevalences for wild-type gene for *TPSAB1* and for HαT.

A less common cause of BST elevation is systemic mastocytosis (SM), a rare myeloproliferative neoplasm with a prevalence of 1-2/10,000.^8^^,^^9^ Here, elevated BST (in most cases >20 μg/L) is an expression of an increase in the number of mast cells in the bone marrow, skin, and extracutaneous organs. The expansion of mast cells in SM is caused by an activating mutation of the *KIT* receptor tyrosine kinase gene at codon D816, *KIT*^D816V^, in over 95% of patients, and which can be detected in peripheral blood.^10^^,^^11^ Almost universally, patients with coexistence of HαT and SM show BST > 20 μg/L.^12^

HαT is associated with mast cell–activation symptoms such as flushing, angioedema, urticaria, and anaphylaxis; it augments these symptoms in patients with SM.^12^ In addition to these classical mast cell–mediated reactions, patients with HαT may also experience medically unquantifiable symptoms such as neuropsychiatric or cardiovascular symptoms, as well as gastrointestinal, muscle, and/or joint pain.^3^^,^^13^^,^^14^ Scientific reports indicate that these symptoms could also be linked to mast cell activation.^15^, ^16^, ^17^ It has been observed that mature tryptases in mast cell granula form α/β-tryptase heterotetramers (2α/2β), with relevance for symptoms *in vivo.*^18^ Individuals with HαT more likely form these heterotetramers owing to the presence of more α-tryptases. Heterotetramers (2α/2β), but not tryptase homotetramers (2β/2β), are able to activate the protease-activated receptor 2 (PAR2), which is expressed on endothelia, epithelia, neurons, smooth muscle cells, and immune cells.^18^ Patients with HαT display increased mucosal leakage after PAR2 activation, which might contribute to irritable bowel syndrome–like symptoms.^18^ Because mast cells preferentially reside at barrier sites, it is conceivable that α/β-tryptase heterotetramers disrupt epithelial or endothelial barriers and drive inflammatory responses.^19^ Therefore, HαT potentially affects many tissues and organs and may lead to multifaceted mediator-related functional symptoms.

There is a lack of knowledge about associated symptoms, aggravated conditions, and symptom burden related to HαT in allergy patients. Because many of these patients will be seen by an allergist, these experts are in a good position to specifically identify symptomatic HαT individuals by BST screening, thorough history taking, and referral for molecular genetic testing.

The aim of this study was to prospectively evaluate the clinical impact of HαT in patients from an allergy cohort. Symptom burden and symptom spectrum were compared in HαT-positive and HαT-negative patients and with respect to different presenting conditions and sex.

## Methods

### Study design

All patients presenting at the allergy department of the University Medical Center Schleswig-Holstein in Lübeck between July 2022 and June 2024 were routinely tested for BST levels using an ImmunoCAP assay (Thermo Fisher Scientific, Waltham, Mass). If the BST level was ≥8 μg/L, tryptase genotyping and *KIT*^D816V^ mutation testing were performed in blood samples, and patients were invited to participate in the study and complete a symptom assessment questionnaire. The study was conducted in accordance with the Declaration of Helsinki and approved by the institutional review board of the University Medical Center of Schleswig-Holstein (approval 2022-634).

### Genetic analysis

Tryptase genotyping was performed with a customized digital droplet PCR (ddPCR) assay. Copy numbers of α- and β-tryptase genes were determined by *TBSAB1*- and *TBSB2*-specific amplification and probes and *AP3B1* as reference gene. Genomic DNA was purified from peripheral blood buffy coat using the QIAamp DNA Mini Kit (Qiagen, Hilden, Germany). Amplification and ddPCR assays were performed on a QX200 Droplet Digital PCR System (Bio-Rad, Hercules, Calif). The sequences used for α- and β-tryptase–specific primers and probes were adapted from Lyons et al.^3^ High-sensitivity *KIT*^D816V^ testing (limit of detection of 0.009% variant allele frequency) was performed with a customized ddPCR assay using locked nuclear acid probes. Bone marrow biopsy was recommended to patients at risk for SM—that is, in case of *KIT*^D816V^ positivity and/or BST ≥ 20 μg/L.

### Symptom assessment

**Supplementary Figure E1:**
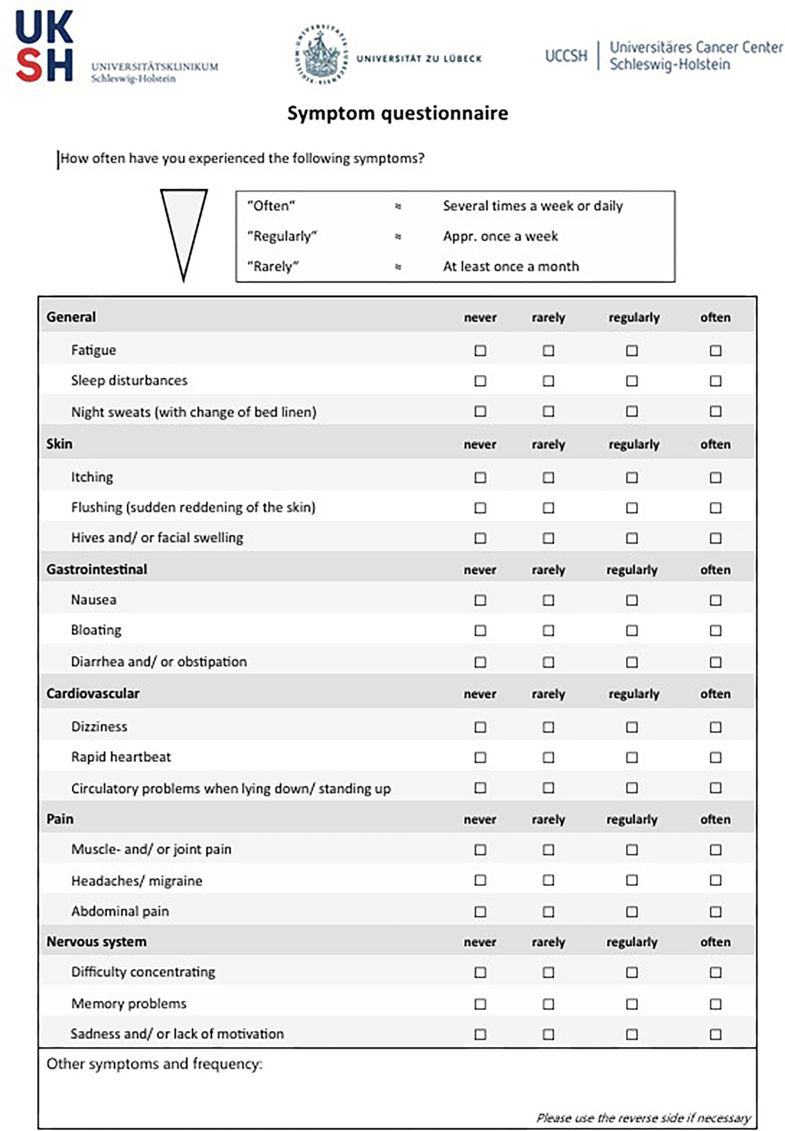


Because there is no clinical validated questionnaire covering both mast cell–related symptoms and functional symptoms associated with HαT, a customized 18-item questionnaire was designed (see Fig E1 in the Online Repository available at www.jaci-global.org). Items were based on the validated mastocytosis quality-of-life questionnaire for SM.^20^ In addition, further mast cell–associated and functional symptoms associated with HαT were included.^12^, ^13^, ^14^ Symptoms were grouped into 6 categories: general symptoms, skin symptoms, gastrointestinal, cardiovascular, pain, and neuropsychiatric symptoms. Each category included 3 specific symptoms. Symptom frequency was assessed as never, rare (once a month, but less than once a week), regular (once a week), and often (several times a week to daily).

### Symptom comparison and control groups

To specifically assess the clinical impact of HαT (and its genotype) and not of BST levels, differences in the symptom burden and pattern of HαT-positive and HαT-negative patients were determined in patients with BST levels indicative of HαT (≥8 μg/L). In support of this approach, a pilot study with 100 patients with BST < 6 μg/L (indicative of HαT negativity) did show a very low extended symptom burden (data not shown).

### Statistical analysis

Descriptive and comparative analyses were conducted by GraphPad Prism v10.3.1 (GraphPad Software, La Jolla, Calif). In multigroup comparisons, appropriate ANOVAs were performed. Differences in BST levels under varying conditions were assessed by comparing means and calculating 95% confidence intervals. Chi-square independence tests were used to exploratively assess differences in symptom prevalence and HαT prevalence between patient cohorts. To evaluate differences in symptom burden (defined as the number of symptoms experienced at least once a week), *t* tests were conducted when normal distribution could be assumed. If normality was not given or group sizes were small (<30 patients), Mann-Whitney *U* test was used to compare median values. The same procedure was applied when comparing BST levels between groups. The significance levels were defined as ∗*P* < .05, ∗∗*P* < .01, and ∗∗∗*P* < .001; #*P* was defined as ≤.1 but >.05. Given the exploratory nature of this study, which aimed to describe clinical features and symptom patterns associated with HαT, no corrections for multiple testing were applied.

## Results

### Study cohort and patient characteristics

**Fig 2:**
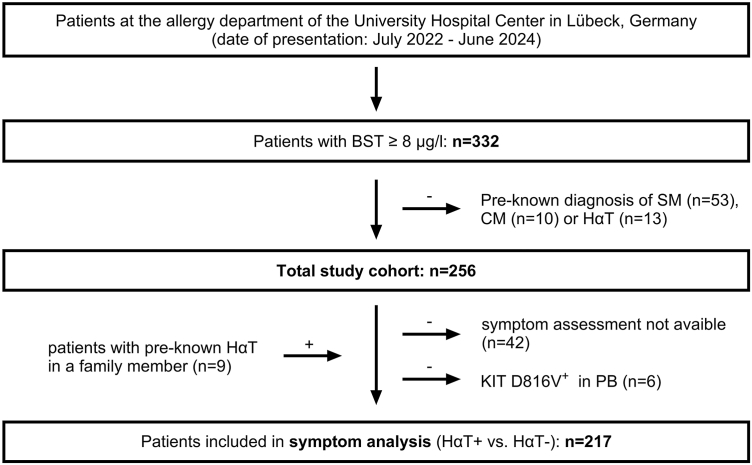
Patient recruitment. Patients presenting between July 2022 and June 2024 were prospectively screened for BST levels; 332 patients with BST ≥ 8 μg/L underwent genetic testing for HαT and *KIT*^D816V^ in PB. Patients presenting with preknown mastocytosis and/or HαT were excluded (n = 256). For symptom analysis and comparison studies, only *KIT*^D816V−^ patients (n = 217) were evaluated. *CM,* Cutaneous mastocytosis; *PB,* peripheral blood.

A total of 332 patients with BST ≥ 8 μg/L were recruited within the study period (Fig 2). To determine the prevalence of HαT and *KIT*^D816V^ and to avoid reporting bias, 76 patients with a preknown diagnosis of SM (n = 53; 46 indolent, 2 smoldering, and 5 advanced SM), cutaneous mastocytosis (n = 10), and HαT/HαT in a family member (n = 13) were excluded. Thus, 256 individuals comprised the study cohort. Of those, 217 (85%) completed the symptom assessment questionnaire and were evaluable for symptom survey.

In total, median age at presentation was 55 years (range, 18-92 years), with slightly elevated median BST levels (12.2 μg/L; range, 8.0-42.0 μg/L; Table I). There was a pronounced female sex predominance (73% female vs 27% male). Urticaria/angioedema was the most common reason for presentation (17%), followed by BST elevation of ≥11.5 μg/L (11%), Hymenoptera venom anaphylaxis (9%), mast cell activation syndrome (9.3%), atopic disease (allergic rhinitis, allergic asthma, and atopic dermatitis; 9%), and drug allergy (9%). Gastrointestinal complaints, food allergy, and idiopathic anaphylaxis were less abundant reasons for initial presentation.

**Table I:** Patient characteristics and reasons for presentation

Characteristic	Total study cohort	Symptom analysis cohort
No. of subjects	256	217
Age (years), median (range)	56 (18-92)	55 (18-86)
BST (μg/L), median (range)	12.2 (8.0-43.0)	12.8 (8.0-39.0)
Sex		
Female	187 (73.3)	163 (75.1)
Male	68 (26.7)	54 (24.9)
Reason for presentation		
Urticaria/angioedema	57 (22.3)	44 (20.3)
BST elevation (≥11.5 μg/L)	37 (14.5)	35 (16.1)
Hymenoptera venom anaphylaxis	31 (12.1)	22 (10.1)
MCAS	31 (12.1)	28 (12.9)
Atopic disease	30 (11.7)	23 (10.6)
Drug allergy	29 (11.3)	24 (11.1)
Gastrointestinal complaints	18 (7.0)	15 (6.9)
Food allergy	7 (2.7)	5 (2.3)
Idiopathic anaphylaxis	7 (2.7)	5 (2.3)
Other	9 (3.5)	7 (3.2)
HαT in family member	—	9 (4.1)

Data are presented as nos. (%) unless otherwise indicated. Patients from total cohort were excluded from symptom analysis cohort if they tested positive for *KIT*^D816V^ in peripheral blood or bone marrow biopsy (ie, SM); or if symptom assessment was not available.

*MCAS,* Mast cell activation syndrome.

### HαT is the most common cause of BST elevation

**Fig 3:**
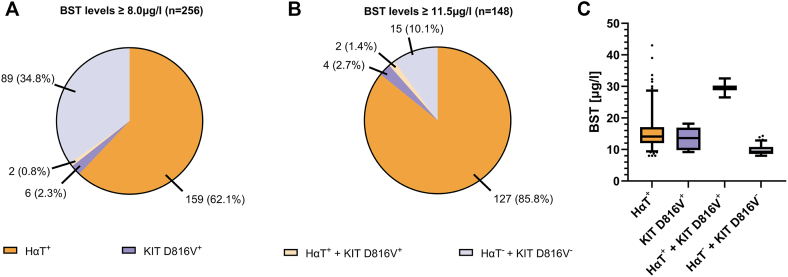
Prevalences and BST levels of patients with HαT and *KIT*^D816V^ mutation. Prevalences of HαT and *KIT*^D816V^ mutation in BST levels **(A)** ≥8 μg/L and **(B)** ≥11.5 μg/L (BST elevation). **(C)** Dot plot comparison (mean and 95% confidence intervals) of BST levels per HαT and *KIT*^D816V^ mutation status.

HαT genotyping and *KIT*^D816V^ mutation detection were performed in all patients with BST ≥ 8 μg/L (Fig 3, *A*). Of 256 patients, 159 (62%) were HαT-positive only, and 2 additional patients (1%) were positive for both HαT and *KIT*^D816V^. The *KIT*^D816V^ mutation was only detected in 6 patients (2%). Five of 8 *KIT*^D816V+^ patients consented to bone marrow examination and SM (indolent SM) was confirmed. Eighty-nine patients (35%) had neither HαT nor a *KIT*^D816V^ mutation. This fraction decreased to 10% (15/148) in the BST levels above the reference value (≥11.5 μg/L). In other words, at BST ranges of ≥11.5 μg/L, HαT^+^ and/or *KIT*^D816V+^ accounted for 9 of 10 cases. HαT (129/148, 87%) was far more common than *KIT*^D816V^ positivity (6/148, 4%) (Fig 3, *B*).

### Absence of HαT demasks *KIT*^D816V^ mutation at BST ≥ 15 μg/L

We next analyzed BST levels according to HαT and *KIT*^D816V^ status (Fig 3, *C*). HαT^+^ patients without *KIT*^D816V^ mutation (n = 159) showed a mean BST level of 15.6 μg/L (range, 8.0-43.0 μg/L). Five HαT patients (3%) had BST > 30 μg/L, with 4 having more than one duplicated α-tryptase copy. Remarkably, all 6 *KIT*^D816V+^ patients had BST < 20 μg/L (mean, 13.5 μg/L; range, 9.2-18.2 μg/L), and thus their disease would not have met the World Health Organization minor diagnostic criterion of BST ≥ 20 μg/L for SM.^21^ HαT^−^ and *KIT*^D816V−^ patients (n = 89) showed significantly lower mean BST levels (9.7 μg/L, *P* < .001), which ranged no higher than 14.3 μg/L. Both patients who were HαT^+^ and *KIT*^D816V+^ displayed BST > 20 μg/L. Taken together, the disease of patients who presented at to our allergy department could not be discriminated into HαT^+^ or *KIT*^D816V+^ by BST levels. In patients with BST ≥ 15 μg/L, all BST increases were attributable to either HαT and/or *KIT*^D816V^. Thus, the absence of HαT at BST ≥ 15 μg/L may demask the presence of the *KIT*^D816V^ mutation.

### Absolute number of HαT patients is highest at BST < 15 μg/L

**Fig 4:**
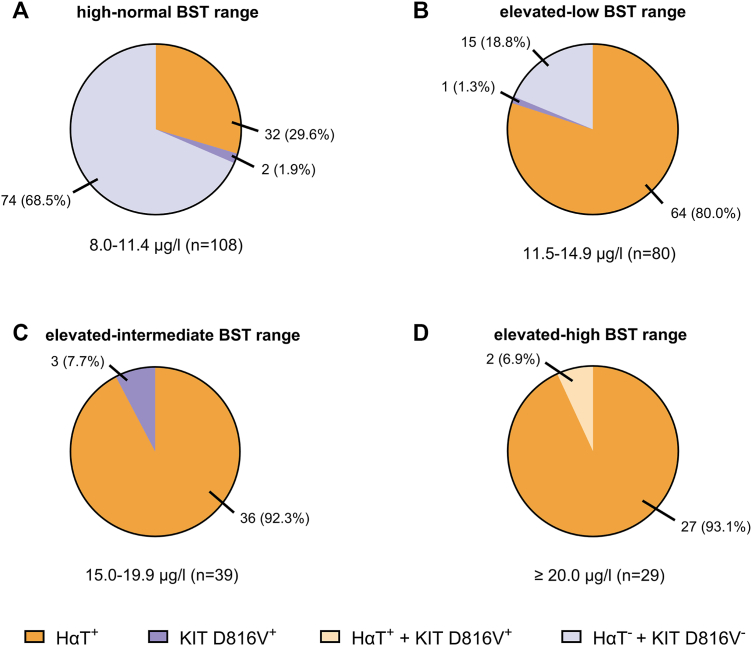
**A-D,** Prevalences of HαT and *KIT*^D816V^ mutation according to defined BST ranges.

Prevalences of HαT varied greatly in the different BST ranges (Fig 4). Although the prevalence of HαT strongly increased to 96% (65/68) at BST ≥ 15 μg/L compared to 51% (96/188) at BST < 15 μg/L, the absolute number of HαT^+^ patients was higher at BST < 15 μg/L (96/161, 60% of all HαT patients). A considerable number of these HαT patients had high-normal BST levels of 8-11.4 μg/L (31/96, 32%), and 68% (65/96) had slightly elevated BST levels of 11.5-14.9 μg/L. *KIT*^D816V^ positivity remained at low prevalence (<10%) at all BST ranges.

### Prevalence of HαT varies according to sex and presenting condition

There was a significant female sex preponderance of patients with the HαT genotype compared to male patients (67% vs 50%; *P* = .021) (see Table E1 in the Online Repository available at www.jaci-global.org). Compared to the prevalence of 63% for all presentation reasons, HαT was enriched in patients presenting for gastrointestinal complaints (77.8%; *P* = .174), in patients with Hymenoptera venom anaphylaxis (74.3%; *P* = .165), and in patients with atopic diseases (73.3%; *P* = .206). Patients presenting with urticaria/angioedema constituted the largest subgroup and exhibited a HαT prevalence of 43.9% (*P* < .001).

### HαT patients show an expanded spectrum of symptoms

**Fig 5:**
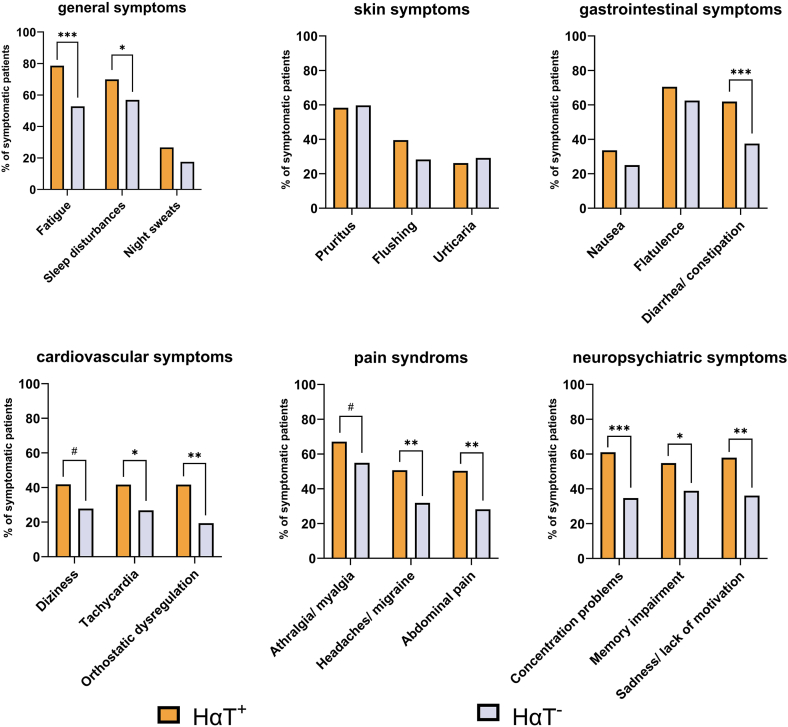
Symptom spectrum in HαT^+^ vs HαT^−^ patients. Frequencies of specific symptoms (reported as at least once a week) were analyzed in HαT^+^ (n = 145) and HαT^−^ (n = 72) patients (all negative for *KIT*^D816V^ in peripheral blood) and indicated per symptom. Chi-square independence tests were performed; significant results are shown as ∗*P* < .05, ∗∗*P* < .01, ∗∗∗*P* < .001, and .05 < #*P* < .1.

Next, we analyzed the symptom spectrum of our patients by assessing the frequency of specific symptoms that occurred regularly—at least once a week (Fig 5). We compared HαT^+^ (n = 145) and HαT^−^ (n = 72) patients, excluding *KIT*^D816V+^ patients. HαT^+^ patients were significantly more affected by pain syndromes such as headaches/migraines (51% vs 31%; *P* = .006) and abdominal pain (50% vs 28%; *P* = .002) compared to HαT^−^ patients. They were also significantly more likely to experience neuropsychiatric impairment, such as concentration problems (61% vs 34%; *P* < .001), memory impairment (55% vs 38%; *P* = .020), and sadness/lack of motivation (58% vs 37%; *P* = .004). General symptoms such as fatigue (78% vs 52%; *P* < .001) and sleep disturbances (70% vs 56%; *P* = .049), cardiovascular symptoms such as tachycardia (41% vs 27%; *P* = .039), and orthostatic dysregulation (41% vs 20%; *P* = .002) were also significantly more abundant in HαT^+^ patients. Interestingly, skin symptoms did not differ between the two groups in our allergy center. Together, individuals with HαT showed an expanded spectrum of symptoms with a marked clustering to pain, as well as neuropsychiatric and cardiovascular symptoms.

### HαT patients display higher symptom burden and symptom dissemination

**Fig 6:**
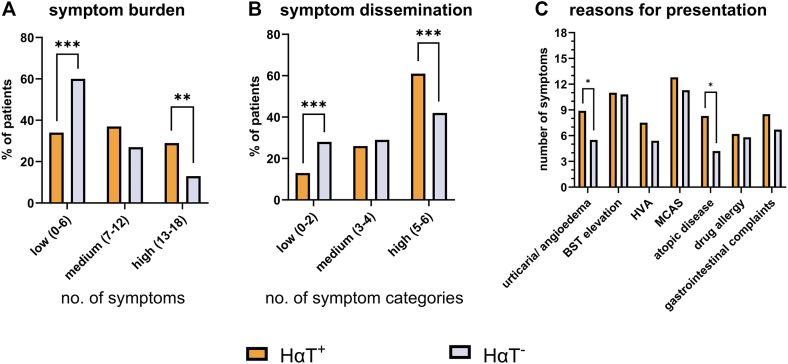
Symptom burden and dissemination in HαT^+^ vs HαT^−^ patients. Comparison of **(A)** symptom burden (number of symptoms reported at least once a week) and **(B)** symptom dissemination (number of symptom categories affecting patients) in *KIT*^D816V−^ patients with HαT (n = 145) and without HαT (n = 72). Chi-square independence tests were performed. **(C)** Comparison of symptom burden of HαT^+^ and HαT^−^ patients according to reason for presentation. Mann-Whitney *U* test was performed. Significant results are shown as ∗*P* < .05, ∗∗*P* < .01, and ∗∗∗*P* < .001. *MCAS,* Mast cell activation syndrome.

To further evaluate our findings, the symptom burden, defined as the total number of symptoms reported at least once a week, was analyzed in HαT^+^ and HαT^−^ patients who were negative for *KIT*^D816V^. Total symptom burden was at 9.3 symptoms in HαT^+^ patients and 6.5 symptoms in HαT^−^ patients (+2.8 symptoms; *P* < .001). Results were further divided into 3 symptom subgroups: low (0-6 symptoms), medium (7-12 symptoms), and high (13-18 symptoms) (Fig 6, *A*). While HαT^+^ patients were more affected by a high symptom burden compared to HαT^−^ patients (29% vs 13%, *P* = .007), HαT^−^ patients were more often represented in the subgroup with no or only a low number of symptoms (60% vs 38%, *P* < .001). Symptom dissemination was evaluated by recording the number of symptom categories (ie, number of organs) affecting patients (at least one symptom within the category; Fig 6, *B*). HαT^+^ patients more often experienced symptoms corresponding to 5 or 6 symptom categories than did HαT^−^ patients (60% vs 41%; *P* = .009).

### HαT significantly affects the clinical phenotype of patients with urticaria and atopic diseases

We further determined the impact of HαT on the symptom burden of patients according to their various reasons for presentation (Fig 6, *C*). HαT^+^ patients presenting for urticaria/angioedema and atopic diseases displayed a significantly higher number of symptoms over HαT^−^ patients of the same presenting reason (respectively, 8.9 vs 5.5, +3.4, *P* = .023; and 8.3 vs 4.2, +4.1, *P* = .016). Of note, the presence of HαT did not significantly affect symptom burden in patients presenting with a history of BST elevation (11.0 vs 10.8, +0.2, not significant) or mast cell activation syndrome (12.8 vs 11.3, +1.5, not significant).

## Discussion

This study substantiates the impact of HαT on clinical symptoms in a large cohort of allergy patients. In HαT patients, the spectrum of manifestations showed to be significantly extended to symptoms such as pain and neuropsychiatric and cardiovascular symptoms. In addition, the majority of patients with HαT (60%) reported symptoms across 5 or all 6 organ systems that were evaluated, and they experienced a significantly higher number of symptoms, with 9.3 compared to 6.5 symptoms in HαT^−^ patients, out of 18 symptoms evaluated. This emphasizes the impact of this genetic trait not only on symptom burden but also on the broad distribution of symptoms across organ categories. The increased symptom burden and extended spectrum of symptoms observed in individuals with HαT might be mediated by α/β-tryptase heterotetramers, which specifically activate PAR2 expressed on endothelia, neurons, smooth muscle cells, T cells, and mast cells.^18^ Tryptase heterotetramers (2α/2β) preferentially form naturally in individuals with HαT and are able, in contrast to homotetramers (2β/2β), to activate PAR2 on cell types such as intestinal epithelial cells.^18^

In contrast to previous findings,^12^^,^^14^ classical mast cell mediator– and allergy-related symptoms such as pruritus, flush, and urticaria were equally distributed between HαT^+^ and HαT^−^ patients in our allergy cohort. This can be explained by the observation that these symptoms are frequently reported by allergy patients. Urticaria/angioedema constituted the most frequent reason for presentation in our cohort. In view of this observation, the prevalence of HαT in these urticaria/angioedema patients of 44% must be considered remarkable, as these patients almost exclusively are primarily referred to an allergy department. These data are underscored by the fact that we demonstrated a significant clinical impact of HαT especially for these patients. Cardiovascular symptoms, fatigue, sleep disturbances, and pain syndromes were significantly more prevalent in urticaria/angioedema HαT^+^ patients than in HαT^−^ patients (data not shown). A subset of urticaria patients has previously been described with elevated BST levels.^22^, ^23^, ^24^ In patients with chronic spontaneous urticaria, increased BST levels were associated with a higher disease burden of systemic complaints.^24^ Our results suggest that HαT might be associated with an extended clinical phenotype in chronic urticaria patients with elevated BST levels. The same could apply to patients with atopic diseases where we detected a significant higher number of symptoms (eg, higher symptom burden) if patients were HαT^+^. Specifically, fatigue, sleep disturbances, and neuropsychiatric symptoms prevailed in these patients compared to HαT^−^ atopics (data not shown). In the latter, neuropsychiatric symptoms were completely absent (data not shown). Together, these data show that in patients presenting at an allergy center, HαT may be associated with extended phenotypes beyond the classical allergy–immunology–driven symptoms.

We noted an enriched prevalence of HαT of 78% in patients presenting with BST ≥ 8 μg/L and gastrointestinal complaints. This observation came unexpectedly because it has been shown that the prevalence of HαT is not increased in irritable bowel syndrome patients compared to the general population.^15^ However, patients presenting with gastrointestinal symptoms to our department previously received extensive assessment by gastroenterologists and did not meet the criteria for irritable bowel syndrome or inflammatory bowel disease as evaluated by dedicated specialists. In addition, the search for IgE sensitizations to food antigens remained predominantly negative in our HαT patients (data not shown). Thus, the enrichment of HαT in these patients may be explained by a HαT-dependent chronic intestinal inflammation, which might be mediated by α/β-tryptase heterotetramer/PAR2–induced leaky gut.^15^ *In vitro,* these heterotetramers were shown to be capable of disrupting intestinal tight junction integrity and of inducing paracellular permeability in a PAR2-dependent manner.^25^ Along this line, examinations of duodenal and terminal ileum samples from HαT patients showed a higher amount and higher activity of mast cells compared to symptomatic patients with Crohn disease or healthy individuals.^15^ Together, these findings should alert allergists for BST screening in patients presenting with functional gastrointestinal symptoms and consecutive referral for HαT testing in patients with BST ≥ 8 μg/L.

In our cohort, HαT diagnostic workup unmasked *KIT*^D816V positivity^ (and SM) in cases with BST levels below the World Health Organization minor criterion of 20 μg/L for SM.^21^ BST ≥ 15 μg/L proved to be a robust marker for HαT and/or *KIT*^D816V^ mutation (Fig 4).

Thus, in patients with BST ≥ 15 μg/L, a negative HαT test result may most likely unmask SM or other myeloid disorders, and bone marrow biopsy should be performed independent of *KIT*^D816V^ mutation status in peripheral blood.

This study demonstrates the substantial clinical impact of HαT in patients presenting to an allergy center. This is particularly true for patients with urticaria/angioedema and atopic diseases, and it might also account for patients presenting with medically preevaluated, nonspecific gastrointestinal symptoms. Further comprehensive research in this field should improve our understanding of complex symptoms in allergy patients and help advance treatment.

**Key messages**

• Physicians should be aware of the potential impact of HαT and assess extended symptoms in allergy patients.
• If symptoms are present, tryptase should be measured and HαT testing initiated at BST ≥8 μg/L.

## Disclosure statement

Disclosure of potential conflict of interest: D. Koch received financial support through the doctoral scholarship “Lübeck Excellence in Medicine” from the University of Lübeck for this research. The rest of the authors declare that they have no relevant conflicts of interest.

## Supplementary data

Supplementary Table E1
