# Supplementary material for: Extended multisystem manifestations of hereditary α-tryptasemia in an allergy center cohort
Source: J Allergy Clin Immunol Glob. 2026 May 20;5(4):100737. doi: 10.1016/j.jacig.2026.100737 (PMC13254390; doi:10.1016/j.jacig.2026.100737)
Supplement: Supplementary Table E1 [file mmc1.docx]

|  | **BST (**µg/l),  median (range) | **HαT prevalence**  No. of HαT^+^ / total no. (%) | **Relative difference**  % (95%-CI) | **p-value**  X2-test/ Fisher-exact-test |
| --- | --- | --- | --- | --- |
|  | | | | |
| Female | 13.3 (8.0-39.0) | 126/188 (67.0) | +15.6% (+2.0, +29.2) | 0.025 |
| Male | 14.4 (8.1-43.0) | 35/68 (51.5) | - 5.6% (-2.0, -29.2) | 0.025 |
|  | | | | |
| Urticaria/ angioedema | 12.8 (8.0-43.0) | 25/57 (43.9) | - 24.6 (–39.0, –10.1) | < 0.001 |
| BST elevation (≥ 11.5 µg/l) | 15.7 (12.0-32.3) | 31/37 (83.8) | +24.5 (+10.9, +38.0) | 0.015 |
| HVA | 16.5 (8.1-33.6) | 23/31 (74.2) | +12.9 (–3.7, +29.5) | 0.165 |
| MCAS | 12.4 (8.0-21.1) | 21/31 (67.7) | +5.5 (–12.1, +23.1) | 0.552 |
| Atopic disease | 14.4 (8.1-32.5) | 22/30 (73.3) | +11.9 (–5.1, +28.9) | 0.206 |
| Drug allergy | 12.2 (8.0-31.5) | 13/29 (44.8) | -20.3 (–39.4, +1.2) | 0.011 |
| Gastrointestinal complaints | 13.3 (9.0-18.4) | 14/18 (77.8) | +16.0 (–4.1, +36.2) | 0.174 |
| Food allergy | 10.9 (10.5-12.6) | 4/7 (57.1) | -5.9 (–43.1, +31.3) | 0.745 |
| Idiopathic anaphylaxis | 10.0 (8.0-12.8) | 3/7 (42.9) | -20.6 (–57.8, +16.7) | 0.263 |
| Others | 11.9 (8.0-20.3) | 5/9 (55.6) | -7.6 (–40.7, +25.4) | 0.652 |
| **all** | **13.6 (8.0-43.0)** | **161/256 (62.9)** |  |  |

**Suppl. Table 1: Median BST level and prevalence of HαT^+^ according to sex and reason for presentation.**

Two patients were positive for HαT and KIT D816V mutation (one female presenting with atopic disease; one male presenting with history of HVA). Relative difference of HαT prevalence indicates the difference between the prevalence of each individual subgroup compared to all remaining patients. BST levels of the two HαT^+^ + KIT D816V^+^ patients were at 32.5 µg/l and 26.5 µg/l, respectively.

*BST = baseline serum tryptase; HVA = Hymenoptera venom anaphylaxis; MCAS = mast cell activation syndrome, MCAS = mast cell activation syndrome*
